# 

**Published:** 1860-02

**Authors:** 


*New Publications Received.*

*Therapeutics and Materia Medica.* A Systematic Treatise on the Action and Uses of Medicinal Agents, including their Description and History. By Alfred Stillé, M. D., late Professor of the Theory and Practice of Medicine in the Medical Department of the Pennsylvania College, etc. In two volumes. Philadelphia: Blanchard & Lea, 1860.

*A Practical Treatise on Fractures and Dislocations.* By Frank Hastings Hamilton, M. D., Professor of Surgery in the University of Buffalo. Surgeon to the Buffalo Hospital of the Sisters of Charity, etc. Illustrated with two hundred and eighty-nine wood-cuts. Philadelphia: Blanchard & Lea, 1860. From the Publishers.

*Elements of Medical Jurisprudence.* By Theodoric Romeyn Beck, M. D., LL. D., Prof. of Materia Medica in the Albany Medical College, etc., and John B. Beck, M. D., Prof. of Materia Medica and Medical Jurisprudence in the College of Physicians and Surgeons of the city of New York, etc. Eleventh edition, with notes by an association of the friends of Drs. Beck. The whole revised by C. R. Gilman, M. D., Prof. of Medical Jurisprudence in the College of Physicians and Surgeons of New York. In two volumes. Philadelphia: J. B. Lippincott & Co., 1860.

*Introductory Lectures and Addresses on Medical Subjects*, delivered chiefly before the Medical Classes of the University of Pennsylvania. By George B. Wood, M. D., LL. D., President of the American Philosophical Society; President of the College of Physicians of Philadelphia; Prof. of the Theory and Practice of Medicine, and of Clinical Medicine, in the University of Pennsylvania, etc. Philadelphia: J. B. Lippincott & Co., 1859. From the publishers.

*The Transactions of the American Medical Association. Instituted*, 1847, Vol. XII. Philadelphia: Printed for the Association. Collins, printer, 705 Jayne St., 1859.

*Proceedings of the American Pharmaceutical Association* at the Eighth Annual Meeting, held in Boston, Mass., Sept., 1859, with the constitution and roll of members. Boston: Press of Geo. C. Rand & Avery, 3 Cornhill, 1859.

*Description of a Deformed, Fragmentary Human Skull*, found in an ancient quarry-cave at Jerusalem; with an attempt to determine, by its configuration alone, the ethnical type to which it belongs. B. J. Aitken Miegs, M. D., Prof. of the Institutes of Medicine in the Medical Department of Pennsylvania College, etc.

*Selections from a Report on Ovariotony;* read before the Kentucky State Medical Society at its annual meeting at Louisville, April, 1857. By J. Taylor Bradford, M. D., Augusta, Ky.

*Botany as an Ally of Medicine.* A Lecture delivered to the Medical Society of the University of Nashville, Dec. 2, 1859. By George D. Blackie, M. D., A. M., F. B. S. E., Prof. of Botany and Natural History in the University of Nashville, etc.

☞ Prof. Austin Flint has now left New York to enter upon the duties of his chair in the New Orleans School of Medicine. He will return early next spring; and until then, correspondents will please address him at New Orleans.

Our office and residence is still at 111 East 21st Street,––Gramercy Park House when all letters to us should be addressed.

